# The HIV Env Glycoprotein Conformational States on Cells and Viruses

**DOI:** 10.1128/mbio.01825-21

**Published:** 2022-03-24

**Authors:** Connie Zhao, Hongru Li, Talia H. Swartz, Benjamin K. Chen

**Affiliations:** a Department of Medicine, Icahn School of Medicine at Mount Sinai, New York, New York, USA; b Division of Infectious Diseases, Department of Medicine, Icahn School of Medicine at Mount Sinai, New York, New York, USA; Carver College of Medicine, University of Iowa; Albert Einstein College of Medicine

**Keywords:** HIV, envelope, conformation, endocytosis, virological synapse, Env, neutralizing antibodies, protein trafficking

## Abstract

The HIV Env glycoprotein is the surface glycoprotein responsible for viral entry into CD4^+^ immune cells. During infection, Env also serves as a primary target for antibody responses, which are robust but unable to control virus replication. Immune evasion by HIV-1 Env appears to employ complex mechanisms to regulate what antigenic states are presented to the immune system. Immunodominant features appear to be distinct from epitopes that interfere with Env functions in mediating infection. Further, cell-cell transmission studies indicate that vulnerable conformational states are additionally hidden from recognition on infected cells, even though the presence of Env at the cell surface is required for viral infection through the virological synapse. Cell-cell infection studies support that Env on infected cells is presented in distinct conformations from that on virus particles. Here we review data regarding the regulation of conformational states of Env and assess how regulated sorting of Env within the infected cell may underlie mechanisms to distinguish Env on the surface of virus particles versus Env on the surface of infected cells. These mechanisms may allow infected cells to avoid opsonization, providing cell-to-cell infection by HIV with a selective advantage during evolution within an infected individual. Understanding how distinct Env conformations are presented on cells versus viruses may be essential to designing effective vaccine approaches and therapeutic strategies to clear infected cell reservoirs.

## INTRODUCTION

Human immunodeficiency virus type 1 (HIV-1) is a lentivirus that infects CD4 receptor-expressing (CD4^+^) immune cells. Untreated, HIV-1 causes a chronic infection that leads to AIDS, characterized by CD4^+^ T cell depletion that leaves patients vulnerable to opportunistic infections and malignancies (1). The use of effective anti-retroviral therapy (ART) suppresses viral replication to prevent HIV-1 transmission and progression to AIDS (2, 3). However, there remain numerous obstacles to overcome, including developing a vaccine and cure for HIV-1.

The HIV-1 envelope (Env) glycoprotein is particularly important in HIV-1 prevention and treatment efforts because it mediates viral entry into host cells (4). The mature Env consists of three exterior gp120 subunits that bind the target cell receptor (CD4) and coreceptor (CCR5 or CXCR4), as well as three non-covalently associated transmembrane gp41 subunits that mediate membrane fusion (5). Env is derived from a gp160 precursor synthesized, folded, trimerized, and glycosylated in the host cell rough endoplasmic reticulum (ER) and subsequently cleaved by host furin-like proteases in the Golgi apparatus (6, 7). The mature trimers transit to the cell surface and are incorporated into budding virions via the endosomal recycling compartment (8). This mature Env is capable of mediating both cell-free infection and cell-cell infection: in the former, virions released from infected host cells infect non-adjacent, uninfected target cells; in the latter, direct cell-cell connections called virological synapses (VS) mediate direct HIV-1 transmission from an infected host cell to an adjacent, uninfected target cell (9, 10).

In addition to mediating viral entry, Env is the only virus-specific antigen exposed on the surface of virions and infected cells (4). Consequently, it is the primary target of host humoral responses, including neutralizing antibodies and Fc-dependent cell-mediated mechanisms such as antibody-dependent cellular cytotoxicity (ADCC), antibody-dependent cellular phagocytosis (ADCP), and complement activation (11–14). The HIV-1 Env has evolved multiple mechanisms to successfully evade these immune responses, including extensive glycan shielding, sequence-variable loops, and conformational flexibility (11–13, 15). Recent single-molecule fluorescence resonance energy transfer (FRET) imaging studies of HIV-1 Env on virus particles reveal that the mature, unliganded Env also samples multiple conformational states to conceal conserved, immunodominant antigens (16).

Here, we review current data surrounding the HIV-1 conformational states of Env on cell-free virus versus the infected cell surface and examine the influence of endosomal recycling, sorting, and post-translational processing of Env in influencing the states that Env can assume in different sites. We consider evidence that differential regulation of the Env conformational equilibrium on virus particles verses the cell surface is regulated by the host cell biosynthetic pathway that recycles heterogeneous Env forms from the cell surface and specifically sorts mature Env to budding virus particles. A clear understanding of how the Env conformational states present on infected cells and virions may be essential in the future development of HIV-1 vaccines and therapies.

## ENV BIOSYNTHESIS AND THE PATHWAY TO THE VIRUS PARTICLE

During infection, Env is synthesized in the late stage of the virus life cycle on the rough endoplasmic reticulum as a polyprotein precursor from a singly-spliced viral mRNA. The nascent Env contains a signal sequence at its N-terminus that targets the cotranslational insertion of the protein into the ER and is removed by a signal peptidase resulting in a type I membrane topology with the N-terminus inserted in the lumen of the ER. Variations in the signal peptide (SP) can influence the glycosylation of Env and its ability to bind to antibodies (17). It is proposed that the SP remains attached to the nascent gp160 in the ER and is cleaved before delivery to the Golgi apparatus (18, 19). Interestingly, the substitution of SP processed with greater efficiency can result in under-processed glycans and altered structure and antigenicity (20).

The Env glycoprotein is heavily glycosylated with around 30 N-linked glycosylation sites modified within the ER-Golgi system by many cellular enzymes. Glycan represents up to half of the mass of Env and occupies 50% to 70% of the surface of Env and thus has a major effect on the antigenicity of the protein. The proper folding of Env and trimerization requires that correct disulfide bonds be made, and interactions with chaperones may facilitate this process. The dense glycan shield contributes greatly to the heterogeneity of the recognized structures on the cell and virion surface. The dense network of high mannose glycans at the surface is often referred to as a glycan shield, decreasing the overall affinity of antibodies that target Env (21–23). Many broadly neutralizing antibodies are glycan dependent, and the loss or gain of N-linked glycan sites is thought to be a common immune escape mechanism during chronic infection (12, 24).

In the Golgi, gp160 precursor is proteolytically cleaved by furin-like proteases into gp120/gp41, an event that is required for the activation of Env (25–30). Uncleaved Env cannot mediate viral membrane fusion and has been described as assuming a non-native state with a higher degree of processed glycans than seen on cleaved gp120 (31). Not all Env is cleaved before trafficking to the cell surface, and a large fraction of Env that is in the cell is uncleaved (32). However, a selective sorting mechanism supports preferential packaging of Env into virus particles which occurs at the plasma membrane where virus particles are formed (33).

The cell surface density of Env is maintained at very low levels by clathrin-mediated endocytic pathways (34). When at the cell surface, a membrane-proximal tyrosine motif, YXXL, is recognized by adapter protein 2 (AP-2) to recruit the clathrin machinery that mediates rapid endocytosis of Env from the surface (35–37). Perturbations of this endosomal internalization signal can alter the efficiency with which individual broadly neutralizing antibodies can block cell-to-cell transmission of HIV (38).

## ENV CONFORMATIONAL STATES

Historically, one of the most significant challenges in structural biology has been developing a three-dimensional structural model for the HIV-1 Env glycoprotein, as its membrane association, conformational heterogeneity, and structural instability initially resisted crystallographic approaches (5, 39). Biophysical studies have suggested that the Env machinery is metastable, transitioning through different conformational states to enable receptor-driven membrane fusion (5). Env binding to target cell CD4 receptor triggers dramatic restructuring of Env from a “closed” to an “open” conformation that exposes the gp120 coreceptor binding site (CoRBS) as well as critical gp41 elements such as the heptad repeat 1 (HR1) coiled-coil and the fusion peptide (FP). Subsequent binding of the target cell coreceptor (CCR5 or CXCR4) to Env promotes FP insertion into the target cell membrane, as well as the association of HR1 and HR2 to form the highly stable six-helix bundle (6HB) that is believed to mediate viral-cell membrane fusion (40–42).

**FIG 1:**
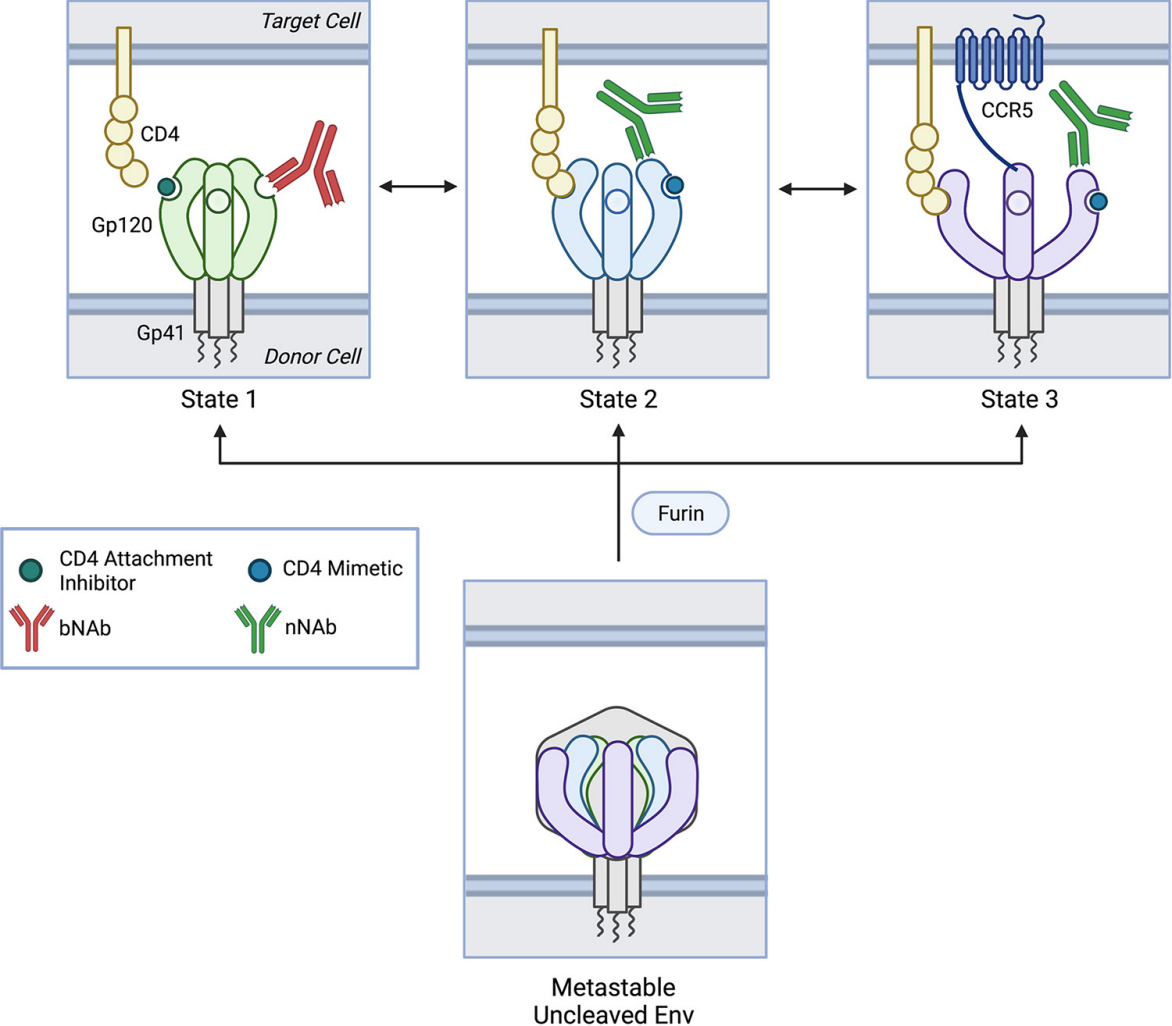
Model of cleaved and uncleaved HIV-1 Env conformational states. The native, cleaved, membrane-associated HIV-1 Env trimer is metastable, occupying an equilibrium of three conformational states that are modulated by CD4 receptor and conformation-sensitive ligands. State 1 or the “closed” conformation is predominant. CD4 receptor binding triggers State 2, an obligate intermediate. The fully “open” conformation, State 3 or the three-CD4-bound conformation, is characterized by coreceptor binding site (CoRBS) formation. State 1 is bound and stabilized by attachment inhibitors and broadly neutralizing antibodies (bNAbs) whereas States 2 and 3 are favored by CD4 mimetic compounds (CD4mc) and non-neutralizing antibodies (nNAbs). The uncleaved Env trimer is similar metastable but favors States 2 and 3. Created with Biorender.com.

Recent single-molecule fluorescence resonance energy transfer (smFRET) studies have demonstrated that the native, mature, membrane-associated Env trimer is, in fact, inherently dynamic. In its unliganded form, Env spontaneously transitions between three distinct, intrinsic prefusion conformations as depicted in Fig. 1: the “closed” State 1, the obligate intermediate State 2, and the “open” State 3 (43, 44). The relative free energies of these conformational states dictate their occupancy in Env’s dynamic conformational equilibrium (44).

***State 1*** represents the predominant conformational state of the native, mature, unliganded membrane-associated Env trimer on the virus particle (43) (Fig. 1). In this conformation, the gp120 variable loops 1 and 2 (V1/V2) obscures most of the V3 loop at the trimer apex (43) while crucial structural elements such as the CoRBS and the HR1 coiled-coil are concealed.

***State 2*** represents an obligate intermediate conformation between States 1 and 3 (43) (Fig. 1). It likely encompasses a set of related conformations that reside in a local energy well in the Env trimer conformational landscape and are therefore accessed during the structural changes between States 1 and 3 (44). CD4 receptor binding stabilizes the downstream, lower energy states, leading to time-dependent progression from State 1, through State 2, to State 3 (45).

***State 3*** represents a more open three-CD4-bound Env trimer conformational state (43, 45–47) (Fig. 1). CD4 binding induces several structural changes such as movement of the V1/V2 loops from the trimer apex to the periphery (43, 46–52), reordering of the gp120 beta sheet bridging domain (46, 50, 52–54), CoRBS formation (46, 47, 55–58), and gp41 HR1 coiled-coil assembly at the trimer axis (46, 47, 52, 59–61). HIV-1 Env trimers that favor State 3 can mediate infection of target cells with low or no CD4 expression (44, 53, 62–70).

Notably, uncleaved Env (71) and Env with gp41 C-terminal tail truncation (ΔCT) (51, 54, 68, 72, 73) tend to sample States 2 and 3 more readily than mature, cleaved, membrane-associated Env. Similarly, all of the current high-resolution HIV-1 Env structures including BG505 SOSIP.664 gp140 complexed with various bNAbs and/or soluble CD4 (46, 47, 51, 54), JR-FL ΔCT complexed with bNAb PGT151 (73), and aldrithiol-2 (AT-2)-treated BaL (74) appear to predominantly occupy States 2 and 3 (45, 74). Therefore, the structure of State 1 remains unknown.

Env may also access off-pathway conformational states such as ***State 2A*** where membrane-associated CD4 interacts with Env to induce exposure of cluster A epitopes in the gp120 inner domain C1 to C2 region. These epitopes are targeted by anti-cluster A antibodies that mediate the majority of ADCC (75). Envs that favor States 2 and 3 (e.g., uncleaved Env) also tend to sample State 2A more readily (76).

The class of antiretrovirals that act as entry inhibitors has expanded recently to include conformation-sensitive attachment inhibitors (also known as conformational blockers), CD4 mimetic compounds (CD4mc). Other Env targeted antagonists include broadly neutralizing antibodies (bNAbs), and poorly or non-neutralizing antibodies (nNAbs), which can also be conformationally sensitive. Conformation-sensitive ligands are unique in that they bind Env and alter the relative free energies and/or activation barriers of the conformational equilibrium in order to stabilize certain states along the entry pathway (43, 44). They not only interfere with the first step of the viral life cycle, but also have the potential to stabilize Env in conformational states that may theoretically present a simplified antigenic target for evolving host immune responses.

***Attachment inhibitors*** are small molecules that inhibit CD4-induced conformational changes at lower concentrations and CD4 binding at higher concentrations (50, 77–83). They reversibly bind to a gp120 pocket comprised of the α1-helix and the β20-21 hairpin adjacent to the CD4 binding loop (77, 84). The prototypical attachment inhibitor is fostemsavir (also known as BMS-663068), which was approved by the United States Food & Drug Administration (FDA) in July 2020 for the treatment of adults with multidrug-resistant HIV-1 infection (85). Temsavir is the active metabolite of fostemsavir, BMS-525629 and other molecules BMS-378806, 484, and 18a are described as having related properties (53, 79, 86, 87). Attachment inhibitors stabilize State 1 (Fig. 1) and, therefore, potently neutralize Env that predominantly occupies State 1, such as mature, cleaved, membrane-associated Env (43–45, 53, 87, 88). In contrast, Envs that favor States 2 and 3 tend to resist neutralization by attachment inhibitors (44, 53).

**FIG 2:**
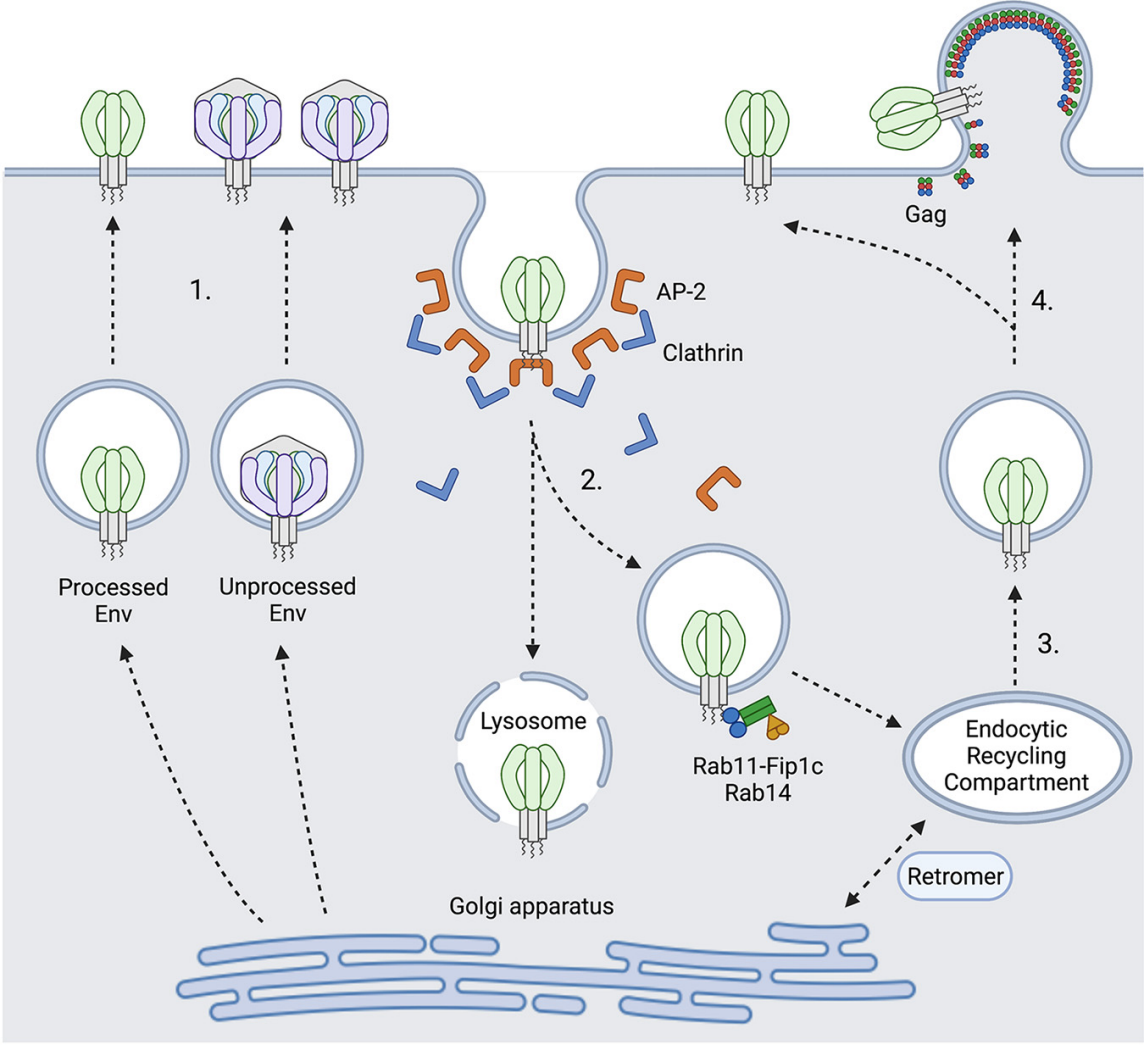
HIV-1 Env trafficking, recycling, and egress. (1) Env trafficking to plasma membrane after synthesis and transit through endoplasmic reticulum and Golgi apparatus. Both fully processed and unprocessed forms of Env can transit to the cell surface. (2) Env endocytosis is initiated by AP2-dependent recruitment of the clathrin endocytosis pathway at the plasma membrane. Early endosomes traffic to recycling endosomes mediated by an interaction of the gp41 cytoplasmic tail with Rab11-FIP1C and Rab14. Alternative pathways for lysosomal degradation of a fraction of endocytosed Env are proposed, but not well documented. (3) Endocytosed Env sorted for outward trafficking or hypothetically could travel to retrograde transport pathways to Golgi apparatus for further proteolytic processing and/or glycosylation. (4) Virus particles are enriched for fully processed Env, which is targeted to the viral assembly site at the plasma membrane. Figure created with Biorender.com.

***CD4mc*** are small molecules the bind the well-conserved gp120 Phe 43 cavity near the CD4 binding site (52, 63, 89, 90). They inhibit Env-mediated fusion and entry through multiple mechanisms, including competitively inhibiting CD4 binding (91), increasing gp120 shedding (65), and triggering a transient, premature, activated conformational state similar to that induced by CD4 that spontaneously and irreversibly inactivates (50, 62, 64, 65). Examples include NBD-556, YYA-021, the DMJ compounds, and BNM-III-170 (100-103). Because CD4mc stabilizes State 2 and 3 (Fig. 2), Env that predominantly occupies State 1 is relatively resistant to neutralization by CD4mc (88, 92). Conversely, Envs that favor States 2 and 3 are highly susceptible to neutralization by CD4mc (43, 44, 53, 65, 70, 87, 90). CD4mc can also stabilize State 2A in combination with certain nNAbs or HIV-positive human sera (76, 93–98).

***BNAbs*** can bind heavily shielded, conserved epitopes on the surface of the “closed” State 1 conformation and can neutralize a wide range of HIV-1 strains (11, 99–101). They rarely occur in natural HIV infection, usually require several years to develop (102–104), and have proven quite challenging to elicit through vaccination (105). Common epitopes include the CD4 binding site (CD4bs), the gp120-gp41 interface, the membrane-proximal external region (MPER), and quaternary and/or glycan-dependent epitopes in the V1V2 and V3 domains (106). Envs that predominantly occupy State 1 are highly susceptible to neutralization by bNAbs (32, 43–45) (Fig. 1), while Envs that favor States 2 and 3 are relatively resistant (44, 66, 68, 70, 107).

***NNAbs*** preferentially recognize non-State 1 conformational states with narrow to no ability to neutralize HIV-1 strains. They appear readily elicited during natural HIV-1 infection and by animal or human Env protein immunization (11, 32, 100, 101, 108–119). Common epitopes include the CD4-induced (CD4i) or CoRBS and V3 epitopes (100, 101, 109). While nNAbs poorly neutralize Envs that predominantly occupy State 1 (50, 77, 80, 82, 88), they are able to potently neutralize Envs favoring States 2 and 3 (43, 44, 50, 53, 66–70, 87, 90, 107, 110, 120–126) (Fig. 1). Anti-cluster A and CoRBS nNAbs can stabilize State 2A in combination with membrane-associated CD4 or CD4mc (76, 93–98). NNAbs also can mediate ADCC, ADCP, and complement activation if they can detect relevant forms of Env displayed on the cell surface. Non-neutralizing responses can also impart significant selective pressure on viral evolution and have been associated with protection in several animal models and one human vaccine clinical trial (127–129).

Overall, the HIV-1 Env trimer is far more dynamic and heterogeneous than initially anticipated, occupying an equilibrium of conformational states dictated by each Env’s intrinsic conformational landscape. This energy landscape is in turn modulated by receptor and coreceptor binding, as well as conformation-sensitive ligands. The ability of a ligand to neutralize an Env trimer depends on its binding affinity as well as the conformational state equilibrium of the specific Env (130). Conversely, the conformational stability or instability of Env can influence its resistance to neutralization (43).

### HIV-1 cell-to-cell infection.

HIV-1 infection can be initiated efficiently by cell-free virus particles or by infected cells through virological synapses (VS), which mediate efficient cell-to-cell infection *in vitro* (131–133). For the purposes of this review, we focus on the role of T cell-to-T cell infection in comparison with cell-free viral infection, to better understand how antibody responses may recognize these modes of infection with variable efficiency. HIV-1 cell-to-cell transmission between T cells and macrophage and trans-infection between dendritic cells and T cells are reviewed elsewhere (134, 135).

The VS is an adhesive structure formed at the cell-cell contact region. It is initiated by interaction between Env on the surface of HIV-infected T cells and CD4 on adjacent uninfected CD4 T cells (9, 131), and is further stabilized by intercellular adhesion molecules including intercellular adhesion molecule 1 (ICAM1), ICAM3 and lymphocyte function-associated antigen 1 (LFA-1) (136). These molecules are enriched at the VS and may enhance VS formation to facilitate cell-to-cell infection (136). Activated leukocyte cell adhesion molecule (ALCAM), which is critical for HIV-1 infected monocyte transmigration through the blood brain barrier in the CNS (137), promotes cell-cell aggregation and VS formation. The loss of ALCAM can diminish cell-to-cell transmission, which can be rescued by restoring cell-cell aggregation through other means (138). A minor allele of ALCAM is reported to be associated with accelerated disease progression (139). VS promotes cell-to-cell infection through dynamic recruitment of viral proteins to the cell-cell contact region (133) with the involvement of cytoskeletal rearrangement (131, 140–142). In addition to supporting transmission between infected and uninfected T cells, cell-to-cell transmission can also occur from infected macrophages to uninfected CD4 T cells (143).

Compared with cell-free infection, cell-to-cell infection demonstrates greater efficiency *in vitro* (9, 144, 145) and evidence for cell-cell infection is supported by *in vivo* small animal models (146, 147). In one model system, Murooka et al. determined that migration of HIV infected T cells from local draining lymph nodes to systemic circulation is required for systemic spread of HIV infection, implying that the dissemination of the infection requires cells as vehicles of dissemination (146). In another humanized mouse study, Law et al. observed that infections that were seeded with HIV-infected cells that were co-infected with two viral genotypes gave rise to newly infected co-infected cells, indicating that the genotypes inherited by newly infected cells is determined by the genotypes present in the input cells (147). In lymphoid tissues where susceptible CD4^+^ T cells are densely packed, immune surveillance creates continual opportunities for cell-cell interactions between infected and uninfected T cells (146, 147). HIV-infected donor cells can interact with multiple adjacent target cells and form polysynapses. The virus transmits through polysynapses at multiple membrane regions simultaneously to facilitate exponential viral growth, potentially contributing to evasion from immune responses (132). Another key feature of HIV-1 cell-to-cell transmission is that multiple copies of viral genomes may be transferred from donor to target cell, resulting in productively infected cells that carry multiple proviruses (147, 148). Multicopy infection may contribute to the virus’s genetic diversity by enabling co-transmission of genetic variants with non-mutant viruses and increasing the opportunity for mutational RT events that occur during each round of infection (149–152). Several studies from Sigal group find that the high copy number of HIV that occurs from cell-to-cell infection can contribute to enhanced resistance to antiretroviral drugs, facilitate persistence in the presence of antiretroviral drugs, and increase the speed of viral gene expression and cell death in infected cells (153–155). Seemingly in contrast to the evidence for multicopy infection seen in experimental models, studies of cells from infected patients have found that the HIV copy number in individual cells from people with HIV have observed that a large majority of cells >90% carry a single copy (156, 157). This may indicate that of those cells that survive or persist with HIV genomes in them, a majority are single copy. Further studies are needed to determine if these single copy infected cells are overrepresented in the cells that survive infection, while cells infected with multiple copies, may produce greater levels of virus experience greater rates of cell death (134, 154).

During chronic HIV-1 infection, circulating virus strains evolve to resist antibody neutralization where mutations allow viral isolates to evade contemporaneous antibody responses *(*12, 158, 159*)*. Individuals that generate abundant high titer neutralizing antibodies against HIV Env have been identified from screens of chronically infected patients (104, 160–163). Because antibodies that can neutralize a broad range of viruses *in vitro* were not isolated from patients in whom viral replication was spontaneously controlled, it can be inferred that the virus replicating in these patients is resistant to the levels of bNAb that are circulating in these individuals (164, 165). The virus has evolved diverse mechanisms to evade and adapt rapidly to immune responses during each infection, including error-prone RNA pol, reverse transcriptase, and innate cellular cytosine deaminases. Cell-to-cell transmission is generally considered as another mechanism by which HIV-1 can evade antibody neutralization (149).

### Cell-to-cell infection is more resistant to bNAb neutralization than cell-free transmission.

The development of single-cell antibody cloning technology led to the discovery of dozens of new generation bNAbs with extraordinary neutralizing breadth and potency (166). These bNAbs have been shown to provide protective immunity against challenges with chimeric simian-human immunodeficiency viruses in macaques and HIV-1 in humanized mice (161, 167–174). Early-stage clinical trials have tested passive infusions of new generation bNAbs, including VRC01, 3BNC117, and 10–1074, and have demonstrated safety and some efficacy (175–178), invigorating interest in antibody therapy for established infections. Evidence of pre-existing resistance to individual bNabs makes it likely that these approaches will require careful consideration of sensitivity and the use of combinations to prevent rapid escape.

In studies specifically focused on cell-to-cell transmission, VS-mediated infection is more resistant to inhibition by antiretroviral agents, HIV-1 patient sera, and new generation neutralizing antibodies compared to the same virus during cell-free infection *in vitro* (38, 155, 167, 179–182) Table 1. This has been shown in the case for both lab-adapted viral strains and viruses with transmitted founder Env (9, 38, 167, 179, 180, 183–186). The differences in IC_50_ between cell-free and cell-to-cell infection are dependent upon viral strain, and the epitopes targeted (38, 183). Discrepancies are observed among different studies regarding the magnitude of differences in IC_50_ of cell-free and cell-to-cell transmission. For example, Abela et al. found CD4bs antibody VRC01 displayed dramatic potency loss while MPER antibody such as 2F5 remained potent against cell-to-cell transmission relative to cell-free infection (184). However, in the study from Duncan et al., MPER antibody 2F5 was much less effective inhibiting cell-to-cell infection over cell-free infection, while CD4bs antibody VRC01 remained equally potent (181). These differences are likely attributable to the use of different viral strains, cell lines, and/or experimental designs (9, 38, 167, 179, 180, 183, 184, 187–192). Despite what may be strain- or cell-specific differences in the study of neutralization of cell-to-cell transmission, in nearly all cases, cell-to-cell infection is more difficult to neutralize than cell-free infection, which supports that it may play an important mechanism for immune evasion. The largest differences in neutralization sensitivity are 2,000- to 3,500-fold less sensitive to neutralization of cell-cell infection relative to infection by cell-free virus. bNabs showing these large differences include 35O22, the gp120/gp41 interface antibody, 10-1074, a V3 glycan targeted antibody, and b12, a CD4 bs antibody (Table 1). The mechanisms that mediate these stark differences in inhibitory potency are still unclear.

**TABLE 1:**
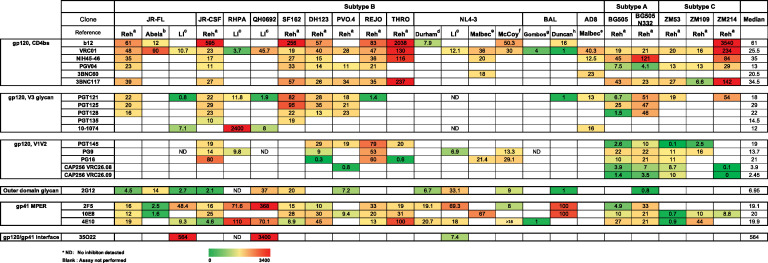
Fold change in half maximal inhibitory concentration (IC50) of neutralizing antibodies comparing cell-free versus cell-to-cell infection

a Reh et al., 2015 (183).

b Abela et al., 2012 (184).

c Li et al., 2017 (38).

d Durham et al., 2012 (179).

e Malbec et al., 2013 (167).

f McCoy et al., 2015 (193).

g Gombos et al., 2015 (180).

h Duncan et al., 2014 (181).

Relative resistance of cell-to-cell neutralization over cell-free infection is reflected as increased IC_50_, and reduced efficacy. A previous study with transmitted founder (T/F) Env carrying constructs in the T cell line system observed incomplete neutralization (38). In many cases, we found an inability of bNAbs to completely neutralize the virus at high antibody concentrations unique to cell-to-cell infection and not seen with infection by cell-free virus. In a severe case, we observed incomplete inhibition where the peak neutralization blocked only 36% of the infection. When a significant fraction of the virus bypasses antibody neutralization through cell-to-cell transmission (38) this is indicative of incomplete neutralization or a decreased efficacy. The Burton group reported an incomplete neutralization phenotype with a very high concentration of highly potent bNAbs (193). They have postulated that the observed incomplete neutralization is partly attributable to the heterogeneity in glycosylation of HIV-1 Env (193). These studies underscore the importance of characterizing the *in vitro* efficacy of bNAbs for their clinical use and vaccine designs, as resistant viral populations would result in failed viral control in patients. The precise mechanisms of how cell-to-cell infection promotes neutralization resistance remain unclear, although one clue reported by Li et al., is that the mutation of a recycling motif in Env, a membrane proximal tyrosine motif YXXL, can have strong effects on neutralization of cell-cell infection, in both positive or negative ways (38). The sensitivity of cell-to-cell transmission mediated by T/F Env QH0692 to b12 (CD4bs Ab) increased in the context of the YXXL mutation, whereas the sensitivity to 35O22 (gp120/gp41 interface Ab) decreased. These results may implicate endocytic recycling in sorting different antigenic forms of Env, or alternatively suggests that the engagement of Env recycling machinery on the intracytoplasmic domain can directly affect Env conformation (38).

### Role of the cytoplasmic tail in modulating Env conformation on cells versus cell-free virus particles.

Recent studies support the hypothesis that cell-associated Env presents a distinct conformation from that presented on cell-free particles, showing different neutralization sensitivities (38, 179, 194). Dale et al. proposed a two-step entry model of HIV-1 cell-to-cell infection where cell-associated virus maturation occurred after viral transfer through the VS, and where viral maturation is required for viral fusion to be activated (194). Based on this model, cell-associated Env is held as a pre-fusogenic conformation during the process of Env-CD4 engagement and the virus material transfer step and is thus likely not well recognized by bNAbs as mature Envs, contributing to neutralization resistance. The study revealed that in VS-mediated, cell-to-cell infection, viral fusion did not occur until cell-associated viral particles were transferred into a trypsin-resistant endocytic compartment of the recipient CD4^+^ cell. It is postulated that Env conformational transitions occur concomitantly with viral maturation from within the endocytic compartment through regulation by the cytoplasmic tail (CT) (194).

In agreement with the notion that the Env CT can control epitope exposure on the cell surface, it is reported that truncation of the cytoplasmic tail of gp41 enhanced neutralization sensitivity in cell-to-cell infection while not in cell-free infection (179). Studies of cell-free viral infection have found that the maturation status of the virus particle, i.e., the proteolytic cleavage of Gag, through interaction with cytoplasmic tail, can control the fusogenic activity of the Env on the particle (72, 195, 196). Cytoplasmic tail truncation mutants exhibit altered neutralization sensitivity compared with wild-type counterparts. These studies support an allosteric model whereby the cytoplasmic tail can regulate the fusogenic potential and the ability of Env to make conformational transitions in the ectodomain of gp120. In support of conformational regulation by the Env CT, both bNAbs and non-neutralizing antibodies are reported to bind to WT cell-surface Envs differently from CT truncation mutants (72).

### Inefficient Env processing and recycling.

While Env that is packaged onto virus particles is typically enriched for fully processed forms, significant levels of uncleaved gp160 Env are present at the cell surface (33, 197) and may present a means by which heterogeneous Env states may be presented on the surface of infected cells. We consider here evidence that a heterogeneous processing state of Env at the cell surface may affect antibody recognition and neutralization.

### Proteolytic cleavage affects Env conformations.

Env trimers once synthesized, glycosylated and proteolytically processed can then travel to the plasma membrane via the secretory pathway and are rapidly recycled from the cell surface into the endocytic compartment to avoid immune detection (Fig. 2) (198–202). Early studies observed that only 5% to 15% of intracellular gp160 was processed into mature gp120, which is then transported to the cell surface and incorporated into virus particles (203). Cleavage deficient gp160 was also found to be directed to the surface of infected T cells (197). Proteolytic processing significantly affects epitope presentation of cell surface Env, alters neutralizing and non-neutralizing antibody recognition, and restrains conformational flexibility (130, 204–206). Zhang et al. observed that the uncleaved gp160 is heterogeneous in its extent of modification by complex carbohydrates (33). A larger population of uncleaved Env without complex glycans cross-links into diverse oligomeric forms, and can be recognized by poorly neutralizing antibodies. smFRET studies show that uncleaved precursor gp160 was the most extensively characterized by smFRET (207) and can sample all three conformational states, displaying a high level of conformational flexibility (45, 71), and shifting more toward state 2 and 3 conformations, resulting in increased binding to non-neutralizing antibodies (71, 204–206). Considering its presence on the surface of infected cells, uncleaved gp160 could distract the immune system so that only non-neutralizing epitopes are present, protecting functional pre-triggered state 1 conformation from being recognized (33).

Uncleaved Env and mature cleaved Env have different antibody binding profiles; most bNAbs preferentially bind to cleaved Env (204), while the uncleaved Env shows increased binding to non-neutralizing antibodies (71, 204–206). Assuming a conformation not recognized by bNAbs, the uncleaved Env at the cell surface may limit recognition, providing a mechanism for resistance of antibody neutralization during HIV-1 cell-to-cell transmission. The heterogeneous characteristics of gp160 on the infected cell surface could also contribute to the incomplete neutralization phenotype during cell-to-cell infection, in which a large fraction of cell-associated virus was not inhibited by bNAbs at saturating concentrations (38).

### Env endocytosis and outward sorting pathway.

HIV-1 infected cells maintain a low abundance of Env on their surface (34). HIV-1 Env is either incorporated into virions or rapidly internalized through Clathrin/AP2 mediated endocytosis (35, 37, 200, 202, 208–210). This is mediated by the tyrosine-based, membrane-proximal sorting motif Y_712_XXL (35, 37, 200–202, 211) and a dileucine motif in the cytoplasmic tail of gp41 (212). The Y_712_XXL motif is highly conserved across HIV-1, HIV-2, chimpanzee simian immunodeficiency virus (SIVcpz) and sooty mangabey SIV (SIVsmm) (211, 213). In addition to constituting a strong endocytic (35, 37, 200, 202) and basolateral sorting signal (214, 215), Y_712_XXL is also required for virion infectivity, viral entry, and can affect Env incorporation in HIV-1 (216). Mutant Env with Y712A packages about 50% of gp120 into virus particles relative to the wild-type counterpart, which correlates with decreased infectivity (216). The virions with Y712A were less fusogenic, as demonstrated by reduced syncytia formation (216). The Y_712_XXL motif also contributes to the polarized budding of the virus. Mutants with the Y712A mutation displayed reduced cell-to-cell transmission efficiency (214). Compared with wild-type Env, the Y712A mutant expressed not only a higher level of Env on the surface of infected cells but also showed differential antibody binding and neutralization sensitivity by bNAbs (38). Studies of the role of the Y_712_XXL motif in SIV suggested that this motif is dispensable for *in vitro* viral replication but is critical *in vivo*. Loss of the Y_712_XXL motif resulted in significant reduction of peak viral load and delayed disease progression in rhesus macaques (217). SIVmac239 with a disrupted YXXL motif by a ΔGY mutation did not deplete mucosal CD4 T cells when infecting rhesus macaques, but was still able to induce immune activation and progression to AIDS (218). Interestingly, pig-tail macaques were able to control replication of SIVmac239 with the ΔGY mutation and preserve mucosal CD4 as well (219), suggesting that the YXXL motif can alter pathogenesis of SIVmac239 in a species-specific manner. A recent NMR structure of the transmembrane segment of gp41 placed the Y_712_XXL motif within the hydrophilic core and demonstrated interaction of Y712 and P714 on one protomer with L704 and V708 on an adjacent protomer respectively, contributing to Env trimer stability (220).

Studies from the Spearman group showed that the outward trafficking of Env from endocytic recycling compartment (ERC) to the virus assembly area is mediated by host factors Rab11-FIP1C and Rab14 (Fig. 2). A tyrosine-based motif Y_795_W in the cytoplasmic tail of gp41 (210, 221) was identified to directly interact with the Rab14-FIP1c complex. A key finding from these studies was a pulse-chase experiment that demonstrated that Env’s internalization from the plasma membrane via Y_712_XXL and Clathrin/AP2 into the endocytic recycling compartment is a prerequisite step for Env incorporation via the Rab11-FIP1c/Rab14 pathway. (8, 210, 221). Recent imaging results from Wang et al. find that a surface labeled Env traffics first to an internal compartment before focally accumulating at a VS when cocultured with CD4^+^ uninfected target cells (222).

The mechanism whereby uncleaved Env traffics to the plasma membrane is not completely understood. A recent study from Zhang et al. confirmed that there is abundant uncleaved gp160 present on the surface of HIV-1 infected cells (33). A dual pathway model was proposed in which cleaved and uncleaved Env adopt different conformations and are transported to the cell surface via different routes (Fig. 2). The uncleaved Env fraction bypassed the Golgi apparatus, traveled to the cell surface without being proteolytically processed or fully glycosylated, and was selectively excluded from virus particles. Mature cleaved Env, with less abundance on the cell surface in many cases, is better represented in cell-free virus particles, can be recognized by bNAbs, and presumably comprises the functional Envs (33).

Jolly et al. proposed the retrograde trafficking model in which the C-terminal 100 amino acids of gp41 directly interact with Vps35 and Vps26 of the mammalian retromer complex and modulate retrieval of recycled Env from the endosomal recycling compartment to the Golgi apparatus following endocytosis (223) (Fig. 2). A potential corollary of this model is that uncleaved Env recycled from the plasma membrane could regain access to the trans-Golgi network Env processing that is thought to occur. Additional studies are needed to determine if Env can be additionally modified when trafficking through recycling pathways.

During VS-mediated cell-to-cell HIV-1 infection, Env accumulates at the VS; however, the physical movements of Env that support the enrichment of Env at the VS are not well characterized. The extent to which Env diffuses laterally during recruitment by the VS from surface pools or is concentrated by a secretory pathway that targets the VS warrants further investigation. Based on the model described above (33, 223) cleaved and uncleaved Envs can both be transported to the plasma membrane and form adhesive structures by transient interaction with CD4, triggering VS formation. Cell surface Env protein is recycled through Y712XXL-Clathrin-AP2 into ERC. From there, uncleaved gp160 could also retrograde traffic to the trans Golgi network (TGN). Through unknown cell sorting mechanisms cleaved Env is then selectively packaged onto virus particles. A recent report from the Finzi group suggests that conformation specific antibodies are differentially endocytosed, which raises the possibility that different conformational states are trafficked differently (224). They reported that bNAbs targeting “closed” conformation of Env induces its internalization from surface of cells infected with both lab-adapted and T/F Envs in a dynamin dependent manner. Interestingly, Envs bound to non-neutralizing antibodies are displayed on cell surface for prolonged period of times.

### Additional considerations that may affect the neutralization of cell-to-cell transmission by bNAbs.

**(i) High avidity of Env at VSs.** A plausible explanation for the neutralization resistance of cell-to-cell HIV infection is that high concentrations of cellular entry receptors and cell-associated Env form a high avidity interaction that requires higher concentrations of antibodies to inhibit. However, a previous study (179) showed that cytoplasmic tail truncation mutants of NL4-3 Env that expressed much higher levels of surface Env expression showed enhanced (rather than diminished) cell-to-cell neutralization sensitivity without changing neutralization sensitivity in cell-free infection.

**(ii) Steric exclusion.** Some studies have examined if the synaptic space at the virologic synapses is structurally restrictive, hiding cell-associated Env epitopes in a privileged space that limits the access of neutralizing antibodies. Abela et al. examined pre-CD4 attachment and post-CD4 attachment steps during cell-to-cell transmission and observed that gp120 directed antibodies, CD4bs antibodies in particular, showed significantly decreased efficiency. In contrast, MPER antibodies and fusion inhibitors still maintained potency against cell-to-cell transmission. They suggested that this may be due to a limited time window when neutralizing epitopes were accessible to antibodies during the formation of virological synapses (184). Duncan et al. examined cell-to-cell transmission from infected macrophages to uninfected CD4^+^ T cells and hypothesized steric restriction as a mechanism for the reduced activity of MPER antibody in cell-to-cell neutralization. They reported that macrophage-to-CD4 T cell transmission was susceptible to CD4bs antibodies and glycan or glycan-dependent epitopes such as those targeted by monoclonal antibody PGT121. Interestingly, they observed that MPER Fab antibodies showed enhanced neutralization efficiency to cell-to-cell infection than the full-length counterpart, suggesting that a smaller Fab domain can better inhibit cell-to-cell infection and that the MPER epitope may be particularly susceptible to steric restriction (181).

Other studies provide evidence against this hypothesis. A study from the Jolly group showed that recombinant llama antibody Fc fusion against CD4bs inhibited cell-to-cell infection even better than the original (smaller) 80kD Ilama antibody J3, indicating that it is not limited by the antibody size (191). Malbec et al. observed that bNAbs that effectively inhibited cell-to-cell infection interfere with the formation of donor-target conjugates and cell-to-cell transfer by accumulating at the VS (167). One important observation was that virus particles captured by target cells in cell-to-cell neutralization assays were mostly coated with bNAbs (167). In another study, anti-gp41 MPER antibodies failed to block virological synapse formation, but could still inhibit cell-to-cell infection (190). These findings together support that epitopes presented by cell-associated viruses are generally accessible by antibodies (191).

In this review we describe potential mechanisms by which differential Env sorting, proteolytic processing, glycosylation, interactions with host factors or newly formed Gag lattice, or trafficking through endocytic pathways may collectively modulate the antigenic state(s) of cell-surface Env distinguishing it from virion-associated Env. Further study of how the trafficking of Env may impact its conformation on its way to assembling virus particles at the VS, and how Env is selected for packaging will provide a better understanding for how HIV-1 infected cells evade antibody neutralization by bNAbs and limit antibody-mediated clearance of infected cells. The results may explain how HIV evades immune responses with such high efficiency.
